# No evidence of genome editing activity from *Natronobacterium gregoryi* Argonaute (NgAgo) in human cells

**DOI:** 10.1371/journal.pone.0177444

**Published:** 2017-05-11

**Authors:** Parisa Javidi-Parsijani, Guoguang Niu, Meghan Davis, Pin Lu, Anthony Atala, Baisong Lu

**Affiliations:** Wake Forest Institute for Regenerative Medicine, Wake Forest University Health Sciences, Winston-Salem, North Carolina, United States of America; University of Tasmania, AUSTRALIA

## Abstract

The argonaute protein from the thermophilic bacterium *Thermus thermophilus* shows DNA-guided DNA interfering activity at high temperatures, complicating its application in mammalian cells. A recent work reported that the argonaute protein from *Natronobacterium gregoryi* (NgAgo) had DNA-guided genome editing activity in mammalian cells. We compared the genome editing activities of NgAgo and *Staphylococcus aureus* Cas9 (SaCas9) in human HEK293T cells side by side. EGFP reporter assays and DNA sequencing consistently revealed high genome editing activity from SaCas9. However, these assays did not demonstrate genome editing activity by NgAgo. We confirmed that the conditions allowed simultaneous transfection of the NgAgo expressing plasmid DNA and DNA guides, as well as heterologous expression of NgAgo in the HEK293T cells. Our data show that NgAgo is not a robust genome editing tool, although it may have such activity under other conditions.

## Introduction

The availability of nucleases that recognize long specific target sequences greatly enhances our ability to edit the mammalian genomes. Zinc finger nucleases [1,2] were the first genome editing tools described, and a zinc finger nuclease specific for *CCR5* has been tested in a clinical trial [3]. However, their toxicity [4] and the difficulty in designing sequence specific zinc finger nucleases prevent their wide application. Through resolving the protein–DNA interaction code of the transcription-activator-like effector (TALE) derived from the plant bacterium Xanthomonas [5,6], a TALE nuclease (TALEN) was developed [7]. Although designing sequence specific TALENs is straightforward, elaborate engineering and assembling are required for each TALEN targeting a specific DNA sequence.

Recently, a clustered regularly interspaced short palindromic repeats (CRISPR)/CRISPR-associated (Cas) system was shown to have genome editing activity in bacteria [8]. Subsequently, multiple reports have demonstrated its genome editing activity in human cells [9–12]. Since CRISPR/Cas9 systems use a CRISPR RNA (crRNA) guide to guide Cas9 proteins to target DNA sequences and the specificity is determined by the single guide RNA, this allows easy programming of Cas9 systems. Cas9 protein from *Streptococcus pyogenes* (SpCas9) was most widely used, while Cas9 from *Staphylococcus aureus* (SaCas9) is increasingly used due to its smaller protein size enabling Adeno-Associated Viral vector delivery [13–15]. Cas9 needs a protospacer-adjacent motif (PAM) adjacent to a specific target sequence, which might not always be available for a specific target. In addition, the performance of a specific crRNA guide is difficult to predict due to possible RNA secondary structure interactions of the crRNA and trans-activating crRNA.

Recently short single stranded DNA-guided sequence-specific DNA endonuclease activity was reported in argonaute proteins from *Thermus thermophilus*, *Pyrococcus furiosus*, and *Methanocaldococcus jannaschii* [16–18], as a host defense system by interfering with invading nucleic acids [16]. These argonaute proteins require temperatures above 37°C to be fully active, which may limit their application in mammalian cells. It was recently reported that the argonaute protein from *Natronobacterium gregoryi* (NgAgo, a mesophilic microbe whose enzymes are more likely to be functional at 37°C) had higher genome editing activity than Cas9 in mammalian cells [19]. Since it does not require a PAM motif and the authors reported high efficiency to targets of high guanine-cytosine content, NgAgo could be a useful tool for genome editing.

Here we compared the genome editing activities of NgAgo and SaCas9. We consistently observed genome editing activity from SaCas9, but not NgAgo.

## Materials and methods

### Constructs

Three NgAgo expressing constructs were used in this study. nls-NgAgo-GK was a gift from Chunyu Han (Addgene plasmid # 78253)[19]. The other two NgAgo expressing constructs were made by inserting synthesized cDNA encoding the HA- or Flag-tagged NLS-NgAgo (GenScript, Piscataway, NJ) into the ClaI and BglII sites of pAAV-MCS (Agilent). The codons for NgAgo were optimized for expression in human cells. The N-terminal additional sequence for Flag-tagged NgAgo is *MDYKDDDDK*APKKKRKVGTGG (the Flag-tag is in *italics* and the nuclear localization signal sequence from SV40 T antigen is underlined). The N-terminal additional sequence for HA-tagged NgAgo is *MAYPYDVPDYA**PKKKRKV*GGGSKRPAATKKAGQAKKKKGTGG (the HA-tag is in *italics* and two nuclear localization signals from SV40 T antigen are underlined). The NgAgo peptide is C-terminal to the added tags and nuclear localization signals. The cDNA and protein sequences for the two NgAgos are listed in Figures A to D in S1 File.

Two SaCAS9 based constructs were made by inserting the guide sequences between the two *BsaI* sites of pX601-AAV-CMV::NLS-SaCas9-NLS-3xHA-bGHpA;U6::BsaI-sgRNA (a gift from Feng Zhang, Addgene plasmid # 61591) [13]. Two commonly studied genome editing targets were tested. One target sequence was from the mutated human hemoglobin beta (*HBB*) gene causing sickle cell disease: AGTAACGGCAGACTTCTCCAC**AGGAGT** (the target sequence is underlined and the PAM of **NNGRRT** is in bold; listed is the guide oligo targeting the anti-sense sequence). Another target sequence was from the human interleukin 2 receptor gamma (*IL2RG*) gene whose mutation causes X-linked severe combined immunodeficiency (SCID-X1): TAGTCTGTCTGTGTCAGGAAC**CTGGGT**. The Cas9/sgRNA expression constructs were named pSaCas9-*HBB*-sgRNA1 and pSaCas9-*IL2RG*-sgRNA1 respectively.

A lentiviral vector (pSin-EF2-*IL2RG*-*HBB*-GFP, for making a GFP reporter cell line) was made by inserting the synthesized DNA between the *EcoRI* and *BamHI* sites of pSin-EF2-Sox2-Pur (a gift from James Thomson, Addgene plasmid # 16577). The synthesized DNA sequence is: GAATTC-**TGA**-GGCCACC-ATG-*GAACCCAGGTTCCTGACACAGACAGACTACACCCAGGGAATGAAGAGCAAGCGCCAT*-*ACTCCTGTGGAGAAGTCTGCCGTTACTGCCCTGTGGGGCAAGGTGAACGTGGATTGGCTAGC*-(*EGFP* Codon 2–239)-GGATCC (GenScript, Piscataway, NJ). A stop codon (in bold) is included at the 5’ end to prevent possible unexpected translation through into *EGFP* cDNA. The target sequences for *IL2RG* (*italics*) and *HBB* (*italics* and underlined) were inserted between the start codon (underlined) and the second codon of *EGFP* cDNA. Insertion of the 119 bp target sequences disrupted the reading frame of *EGFP*. Deletions of 3N+2 or insertions of 3N+1 base pairs by genome editing will restore EGFP expression.

An mCherry-MEX3C^659AA^ fusion protein (exclusively cytoplasmic) expression plasmid was used, as described previously [20]. All DNA manipulations were performed according to the published laboratory manual for molecular biology [21].

### EGFP reporter cell line construction

An EGFP reporter cell line was made to facilitate detection of genome editing events by observing restored EGFP expression. To make the lentiviral vector for reporter cell line construction, 5x10^6^ HEK293T cells were seeded in 10-cm dishes in DMEM medium 24 hours before transfection. Then pSin-EF2-*IL2RG*-*HBB*-GFP Plasmid DNA was co-transfected with psPAX2 and pMD2G DNA (9, 6 and 3 μg respectively) into the cells mediated by 54 μl Fugene 6 according to the instructions of the manufacturer (https://www.promega.com/-/media/files/resources/protocols/technical-manuals/101/fugene-6-transfection-reagent-protocol.pdf). Lentiviral vector production and transduction were performed according to published protocol [22]. The viral vector-containing medium was mixed with pre-warmed fresh 10% FBS containing DMEM medium at 1:1 and 2 ml of the mixture was added to HEK293T cells in a well of 6-well plates. To enhance transduction, polybrene was added to the final concentration of 6 μg/ml. 24 hours after transduction, the cells were incubated in DMEM medium containing 10% FBS and 2 μg/ml puromycin for 7 days to kill the cells without viral DNA integration.

### Cas9 and NgAgo expression

Cas9/sgRNA expression was achieved by direct plasmid DNA transfection or AAV transduction (AAV vectors were made from pSaCas9-*IL2RG*-sgRNA1 and pSaCas9-*HBB*-sgRNA1 at the University of North Carolina at Chapel Hill Vector Core). NgAgo expression was achieved by plasmid DNA transfection. For plasmid DNA transfection, HEK293, HEK293T, and HEK293T-derived reporter cells were cultured in DMEM with 10% FBS, and 100 U/ml penicillin and 100 μg/ml streptomycin (ThermoFisher Scientific). The cells were seeded into 24-well plates at a density sufficient to reach 70–80% confluence 18 hours after being seeded: 1.25x10^5^ cells/well for HEK293T cells and 6x10^4^ cells/well for HEK293 cells. When only plasmid DNA was transfected, Fugene 6 (Roche) was used. When guide DNA was transfected, Lipofectamine 2000 (ThermoFisher Scientific) was used. For cells in one well of a 24-well plate, 0.25–0.5 μg plasmid DNA, 100 ng guide DNA and 1.25–2 μl Lipofectamine 2000 were used respectively. DNA and Lipofectamine 2000 were each re-suspended in 50 μl of OPTI-MEM (ThermoFisher Scientific) before mixing. If two guide DNA oligos were used, 100 ng of each guide DNA was used. When co-transfecting the EGFP reporter plasmid (pSin-EF2-*IL2RG*-*HBB*-GFP) and the NgAgo expressing plasmid, the amounts of reporter DNA and NgAgo DNA were 100 ng and 400 ng respectively. For NgAgo and guide DNA transfection, protocols in Gao’s paper [19] were followed. 48 to 72 hours after transfection, the cells were harvested for analysis. Apart from the modifications described above, general procedures suggested by the manufacturers were followed for Fugene 6 and Lipofectamine 2000 mediated DNA transfection.

### Preparation of 5’ phosphorylated single strand guide DNA

5’ phosphorylated single strand guide DNA oligos were synthesized by Eurofins Genomics (Louisville, KY). In some experiments, the guide DNA oligos were further treated by T4 polynucleotide kinase (New England Biolabs) according to the manufacturer’s instructions to ensure 5’ phosphorylation. The sequences of the guide DNA oligos are listed in Table A in S1 File.

### Immunostaining

HEK293 cells grown on coverslips (6x10^4^ cells/well in 24-well plate) were transfected with NgAgo expressing plasmid DNA with Fugene 6 or Lipofectamine 2000 (similar transfection efficiency in our hands). Twenty-four hours after transfection, the cells were fixed in 4% paraformaldehyde/PBS, pH7.4 at room temperature for 30 min, and permeablized with 0.1% Triton X-100/PBS at room temperature for 30 min. The cells were then blocked with 5% fat-free milk and incubated with anti-Flag antibody (Sigma, F3165, 1:2000, diluted in 1% milk in PBS) or anti-HA antibody (ProteinTech, # 51064-2-AP, 1:1000) at room temperature for 1 hour. After washing three times with PBS, the cells were stained with Alexa594 conjugated anti-mouse secondary antibody (for anti-Flag primary antibody) and anti-Rabbit secondary antibody (for anti-HA primary antibodies). Published immunostaining protocol was followed for immunostaining [23]. Cells were mounted in DAPI-containing mounting medium, and observed under an FV10i confocal microscope (Olympus Corporation, Tokyo, Japan). A 60x objective with a numerical aperture of 1.35 was used. The laser intensity and sensitivity parameters for each channel were kept constant for all samples in the same experiment.

### Western blotting

The cells were lysed by 1x Laemmli buffer containing protease inhibitors (0.5mM PMSF and 1x Complete Protease Inhibitor Cocktail; Roche) and phosphatase inhibitors (50mM NaF, 1.5mM Na3VO3). The proteins were separated by sodium dodecyl sulfate polyacrylamide gel electrophoresis (SDS-PAGE) before transferring to nitrocellulose membranes. SDS-PAGE was performed according to published protocol [21]. The following antibodies were used: anti-Flag (Sigma, 1:2000), anti-HA antibody (ProteinTech, 1:1000) and anti-beta actin (Sigma, 1:5000). Horseradish peroxidase (HRP)-conjugated secondary antibodies were purchased from Pierce. Chemiluminescent reagents (Pierce) were used to visualize the protein signals under the LAS-3000 system (Fujifilm).

### Mycoplasma detection

Cultures were tested for presence of mycoplasma using the PCR Mycoplasma Detection Kit from Applied Biological Materials Inc. (Richmond, BC, Canada). Culture medium was collected 48 hours after incubation and used as template for PCR. The presence of PCR products between 370–550 bp were examined by electrophoresis and ethidium bromide staining. PCR mycoplasma detection was performed according to the instructions of the manufacturer.

### Next-generation sequencing and data analysis

Genomic DNA was isolated from cells treated with Cas9 or NgAgo with the QIAamp DNA Mini Kit (Qiagen, Germantown, MD) according to the manufacturer’s instructions. Then the target region was amplified from the genomic DNA by PCR amplification (primers are listed in Table A in S1 File), using the proofreading HotStart ReadyMix from KAPA Biosystems (Wilmington, MA). The PCR products were analyzed by next-generation sequencing using the Illumina NextSeq 500 (Housed in and operated by the Cancer Genomics Shared Resource, the Comprehensive Cancer Center, Wake Forest Medical School). A mid-output 135 million read flow cell was used. Sequences were read from single ends for 150 cycles. 10% PhiX DNA was spiked into the DNA sample to increase sequence diversity. The percentage of Q30 reads was larger than 85%, and 3–5 million reads were obtained for each sample. The frequency and percentage of a specific sequence in each sample was analyzed using PROC FREQ in SAS software (version 9.4, SAS Institute Inc, Cary, NC). The 20 most appearing types of sequences were aligned with Clustal Omega (http://www.ebi.ac.uk/Tools/msa/clustalo/) to visualize insertions, deletions and mutations. To detect insertions and deletions (INDELs), a program was written in SAS software to calculate the distance between two designated sequences located at the 5’ and the 3’ end of each read. The percentages of the 20 most appearing lengths were then calculated.

## Results and discussion

**Fig 1:**
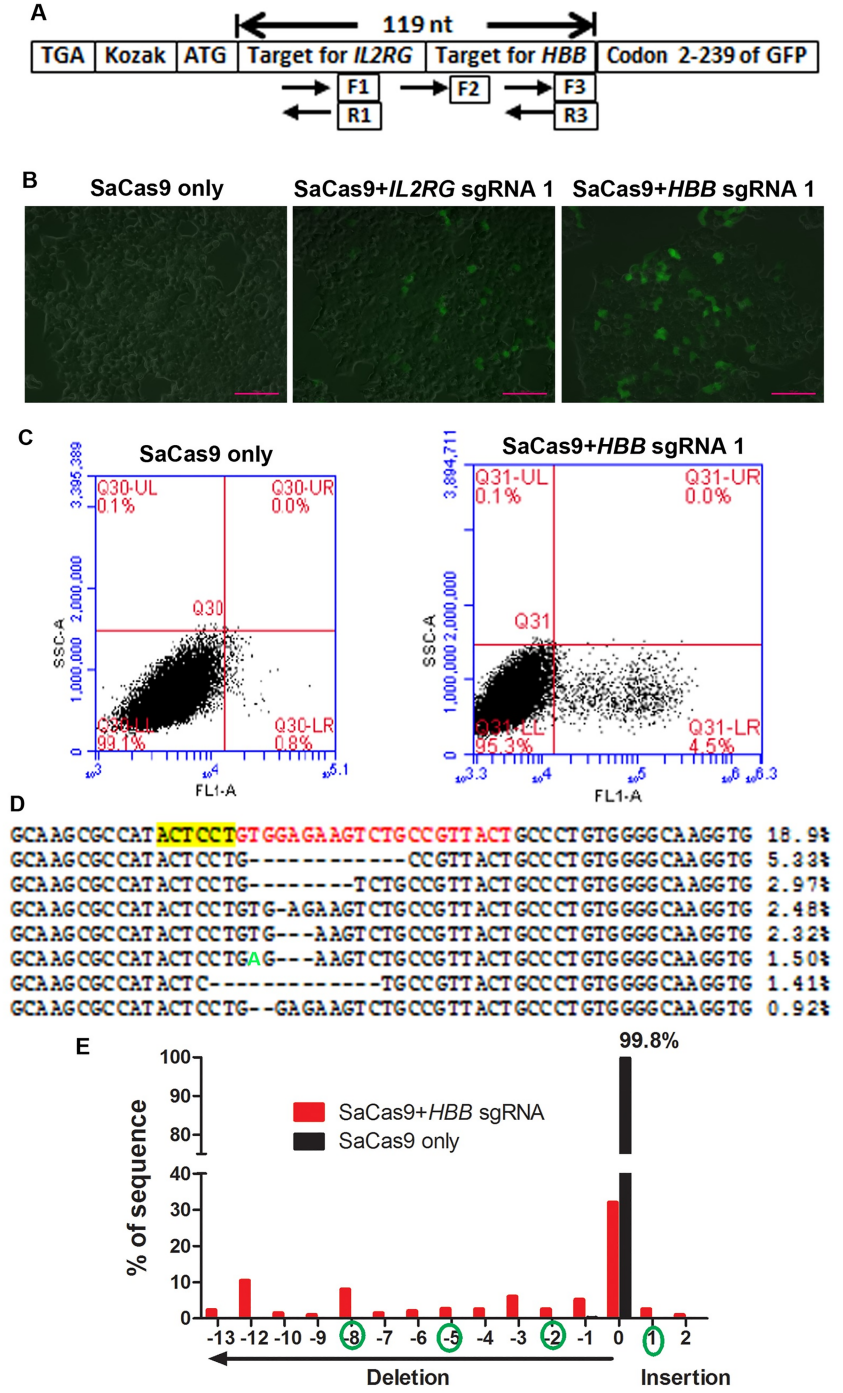
A EGFP reporter cell line for sensitive detection of genome editing activity from SaCas9. **A**. Schematic diagram of components between the start and second codons of *EGFP* cDNA in the reporter cell line. A stop codon was included 5’ to the start codon to prevent unexpected translation into the *EGFP* cDNA from a start codon 5’ to the authentic start codon. A Kozak sequence was included for efficient translation. The inserted 119 bp sequence disrupts the reading frame from the *EGFP* start codon. Deletion of 3N+2 or insertion of 3N+1 base pairs restore the *EGFP* reading frame. Positions and directions (arrow head points to the 3’ end) of the single-strand guide DNA for NgAgo experiments are indicated. **B**. GFP was activated in the presence of SaCas9 and sgRNAs (middle and right) but not without sgRNA (left). Scale bar: 100 μm. **C**. Flow cytometry analysis of EGFP expression 24 hours after DNA transfection of the reporter cells. **D**. Next-generation sequencing analysis of the target region 72 hours after SaCas9 and *HBB* sgRNA1 expression without selection; 3.4 million reads were analyzed. Listed are the most appearing 8 sequences observed. The first sequence (18.9%) has no mutation. The PAM (NNGRRT on the opposite strand) is highlighted yellow and the target sequence is highlighted red. A point mutation is marked green. **E**. Next-generation sequencing analysis of INDEL frequency and size. Types of INDELs that can restore EGFP expression are marked by a green circle.

Since our focus is on the gene editing activities of SaCas9 and NgAgo in human cells, we did not attempt to purify the proteins and test their endonuclease activities *in vitro*. To facilitate the detection of SaCas9 and NgAgo genome editing activity in human cells, we made a stable cell line where enhanced green fluorescent protein (EGFP) expression was disrupted by insertion of a 119-base pairs (bp) sequence (missing 1 bp to make 40 inframe codons) between the start codon ATG and the second codon of *EGFP* cDNA, disrupting the *EGFP* reading frame (Fig 1A). In the 119 bp sequence, we included 57 bp from the human *IL2RG* gene and 62 bp from the human *HBB* gene, which contained SaCas9 target sequences for *IL2RG* and *HBB* respectively. Without genome editing, EGFP will not be expressed due to the insertion of 119 bp which disrupts the EGFP reading frame. This was confirmed by the absence of EGFP-positive cells in the non-transfected reporter cells and in the reporter cells transfected with plasmid DNA expressing SaCas9 without any sgRNA (Fig 1B, left).

After genome editing in the inserted sequence, the double strand breaks are repaired by nonhomologous end joining. Due to the possibility of introducing insertion or deletion (INDELs), deletions of 3N+2 or insertions of 3N+1 base pairs will restore the *EGFP* reading frame and thus EGFP protein expression. To test whether the reporter cells were functional, pSaCas9-*IL2RG*-sgRNA1 (expressing SaCas9 and sgRNA for *IL2RG*) and pSaCas9-*HBB*-sgRNA1 (expressing SaCas9 and sgRNA for *HBB*) DNA was transfected into the reporter cells. 24 hours after transfection, increasing numbers of cells became EGFP-positive (Fig 1B, middle and right images).

Flow cytometry analysis showed that 4.5% to 15% cells became EGFP-positive in pSaCas9-*HBB*-sgRNA1 transfected reporter cells 24 hours post-transfection (Fig 1C). Similar results were observed in reporter cells infected with AAV vectors made from pSaCas9-*IL2RG*-sgRNA1 and pSaCas9-*HBB*-sgRNA1 DNA. We purified the genomic DNA from reporter cells three days after the transduction of AAV made from pSaCas9-*HBB*-sgRNA1, amplified the reporter region by PCR with high-fidelity DNA polymerase, and sequenced the target region by next-generation sequencing. No mutations were found in 18% of sequences. About 80% of sequences had various mutations, and the 20 most appearing mutations were all INDELs (Fig 1D). Our analysis might overestimate the mutation rate caused by SaCas9 genome editing, since some point mutations could be the result of erroneous incorporation during PCR amplification and erroneous call during next-generation sequencing.

We then analyzed the percentage of sequences with INDELs regardless of point mutation. In this analysis, 68% of the reads had INDELs (Fig 1E). Since only part of the INDELs will restore EGFP expression (marked in Fig 1E), and analysis was performed 72 hours after transfection, it is not surprising that we observed lower EGFP-positive rates than INDELs. These data showed that our reporter cells are functional and the SaCas9 system worked in our hands.

**Fig 2:**
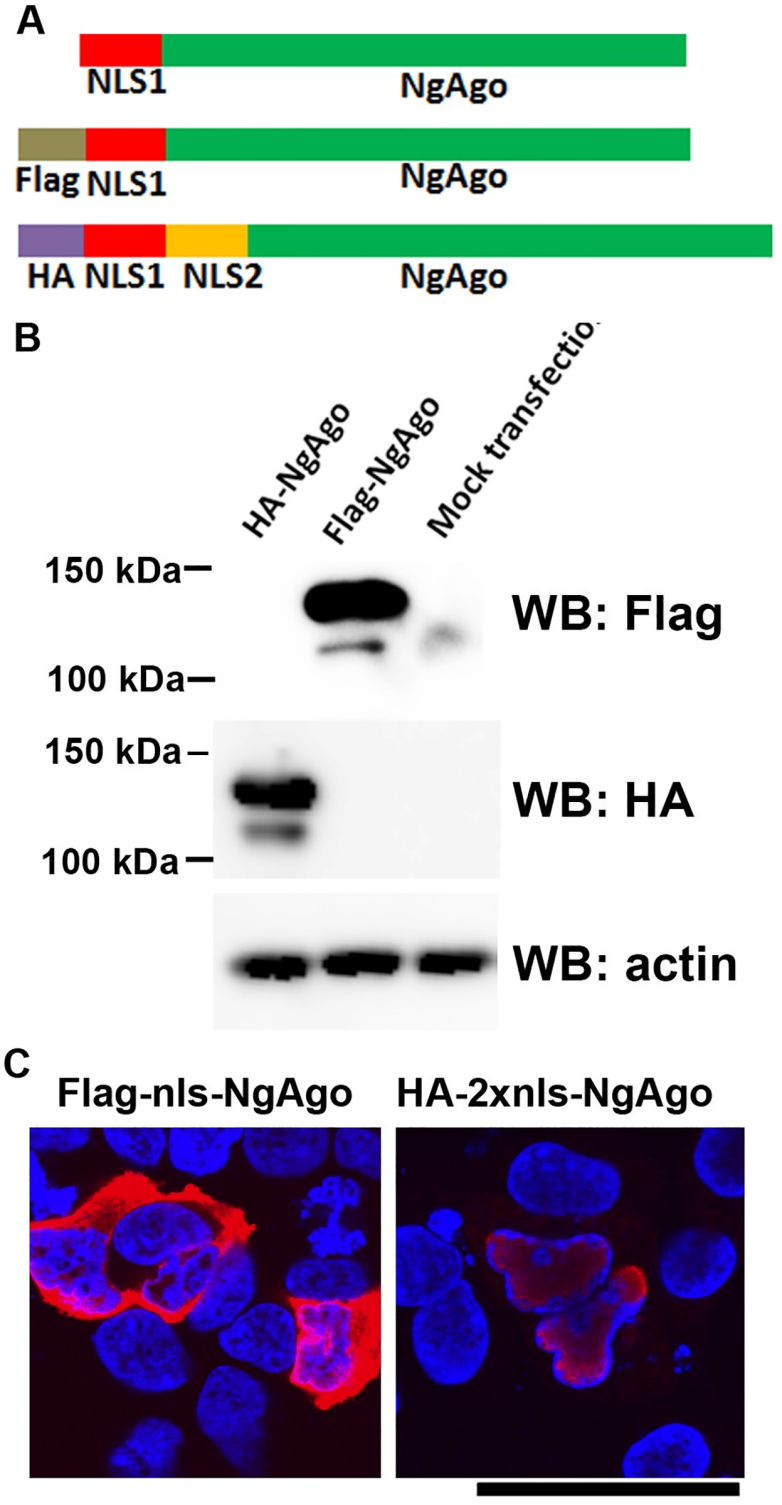
Expression and localization of NgAgo. **A**. Components of the three NgAgo proteins. **B**. Detection of HA- and Flag-tagged NgAgo by Western blots. **C**. Subcellular localization of NgAgo with one NLS signal sequence (Flag-tagged) and two NLS signal sequences (HA-tagged). Red signals were NgAgo (HA or Flag antibody stained, secondary antibody labeled by Alexa594). Nucleus was stained by DAPI and pseudocolored blue. Scale bar: 50 μm.

We used three NgAgo expressing constructs to test the genome editing activity of NgAgo. The construct from Addgene (deposited by the authors of the original NgAgo paper [19]) expresses an untagged NgAgo (Fig 2A, top), and was used for gene editing in human cells in that study. To facilitate detection of NgAgo expression, we also made two constructs expressing Flag- and HA-tagged NgAgo, containing one or two nucleus localization signal sequences from the SV40 T antigen (Fig 2A, middle and bottom). Western blotting showed that both tagged NgAgo proteins were efficiently expressed in HEK293T cells after plasmid transfection (Fig 2B). Immunostaining showed that both tagged proteins could be observed in the nuclei, the NgAgo protein with two NLS signals was exclusive to the nucleus while that with one NLS was visible in both the cytoplasm and the nucleus (Fig 2C). Expression of the untagged NgAgo reported in the original paper [19] could not be tested, since specific antibodies are unavailable.

**Fig 3:**
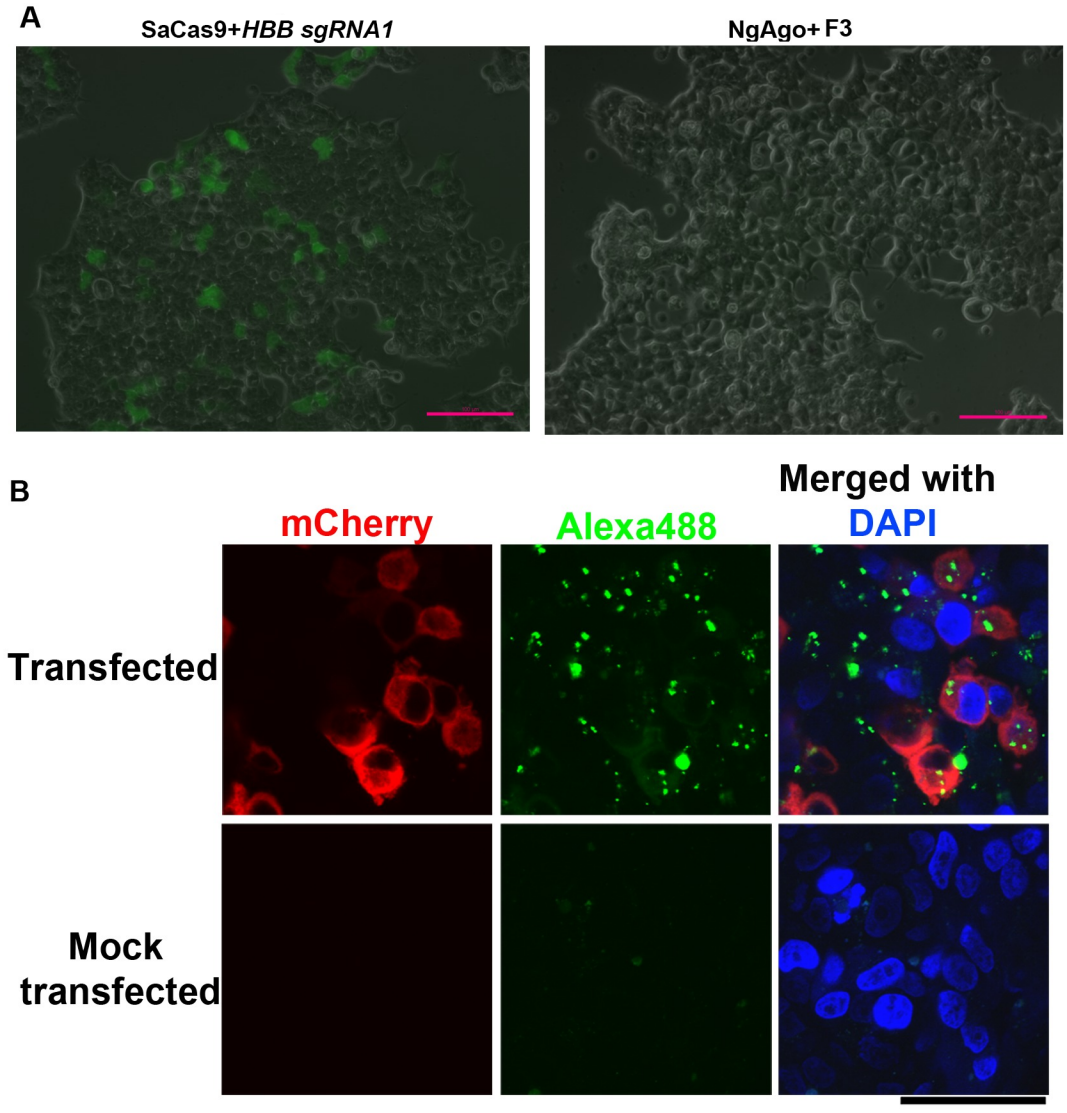
SaCas9 but not NgAgo transfection restores EGFP expression in reporter cells. **A**. EGFP-positive cells were observed in SaCas9 but not NgAgo transfected reporter cells. sgRNA for SaCas9 and 5’ phosphorylated single-strand DNA guide oligo for NgAgo both targeted human *HBB*. A single-strand DNA guide oligo was included in the SaCas9 transfection to equate transfection conditions and exclude possible inhibition of plasmid DNA transfection. **B**. Plasmid DNA and single-strand oligo DNA transfected into reporter cells. Cells were co-transfected with DNA expressing mCherry-MEX3C^659AA^ (a cytoplasmic protein, pseudocolored red) and Alexa488-labeled oligo targeting luciferase cDNA (pseudocolored green). Nucleus was stained by DAPI (pseudocolored blue). Scale bar: 50 μm.

We then tested NgAgo genome editing activity on our reporter cells, which had been shown to express EGFP after being edited by SaCas9. We prepared five 5’-phosphorylated single-strand guide DNA oligos, two of which targeted the same sequences for the *IL2RG* and *HBB* genes as the SaCas9 system (Fig 1A). Although EGFP-positive cells were consistently observed 24 hours after transfection of pSaCas9-*IL2RG*-sgRNA1 or pSaCas9-*HBB*-sgRNA1 DNA, we observed no EGFP-positive cells in any of the NgAgo/oligo transfected reporter cells even after 72 hours (Fig 3A). After we tested all three NgAgo constructs with various 5’ phosphorylated guide DNA oligo combinations (single guide oligo; two guide oligos targeting the same strand or the opposite strands, against the same sequence or different sequences), no EGFP-positive cells could be observed. To rule out the possibility that 5’-phosphorylated guide DNA interfered with transfection efficiency of plasmid DNA, we similarly included guide DNA in pSaCas9-*IL2RG*-sgRNA1 and pSaCas9-*HBB*-sgRNA1 DNA transfections. With or without guide DNA oligos, pSaCas9-*IL2RG*-sgRNA1 or pSaCas9-*HBB*-sgRNA1 DNA transfection generated a similar rate of EGFP-positive reporter cells.

To test whether the transfection conditions we used (the same as reported in the Gao paper [19]) could deliver the plasmid DNA and guide DNA into the cells, we co-transfected Alexa488-labeled luciferase oligo and plasmid DNA expressing MEX3C^659AA^-mCherry (a cytoplasmic protein) [20] into the reporter cells under the conditions used above. 24 hours after transfection, we observed a mCherry fluorescent signal in the cytoplasm of many cells. In addition, punctuated bright green fluorescence signals were visible in mCherry-positive cells (Fig 3B). In lipid-mediated DNA transfection, DNA molecules first enter the endosomes, then escape from the endosomes to function in the cytoplasm or enter the nucleus [24, 25]. Thus expression of MEX3C^659AA^-mCherry indicates successful plasmid DNA entrance into the endosome, escape from the endosome, and subsequent expression of the MEX3C^659AA^-mCherry protein. The bright green fluorescence signals also indicated the presence of Alexa488-labeled oligos in the endosomes. It was difficult to observe oligos already escaped from the endosomes due to their diffuse distribution. However, the escape of the co-transfected plasmid DNA from the endosome suggests the escape of some oligos as well. Similar co-transfection experiments were performed in a recent publication to demonstrate successful transfection of the guide DNA into the cells [26]. Thus, it is highly unlikely that the conditions used in this study were unable to deliver both the plasmid DNA and the single-stranded guide DNA into the cells.

Because it was reported that guide DNA could be loaded into NgAgo at 55°C but not 37°C [19], we thus wondered whether culturing the cells at higher temperatures could yield different results. We heat shocked the reporter cells at 43°C for 3 hours 12 hours after transfection, and then returned the cells to 37°C. The cells survived the heat shock, but EGFP-positive cells were still not observed (data not shown). We did not try higher temperatures since they would damage cells and block the cell cycle [27].

**Fig 4:**
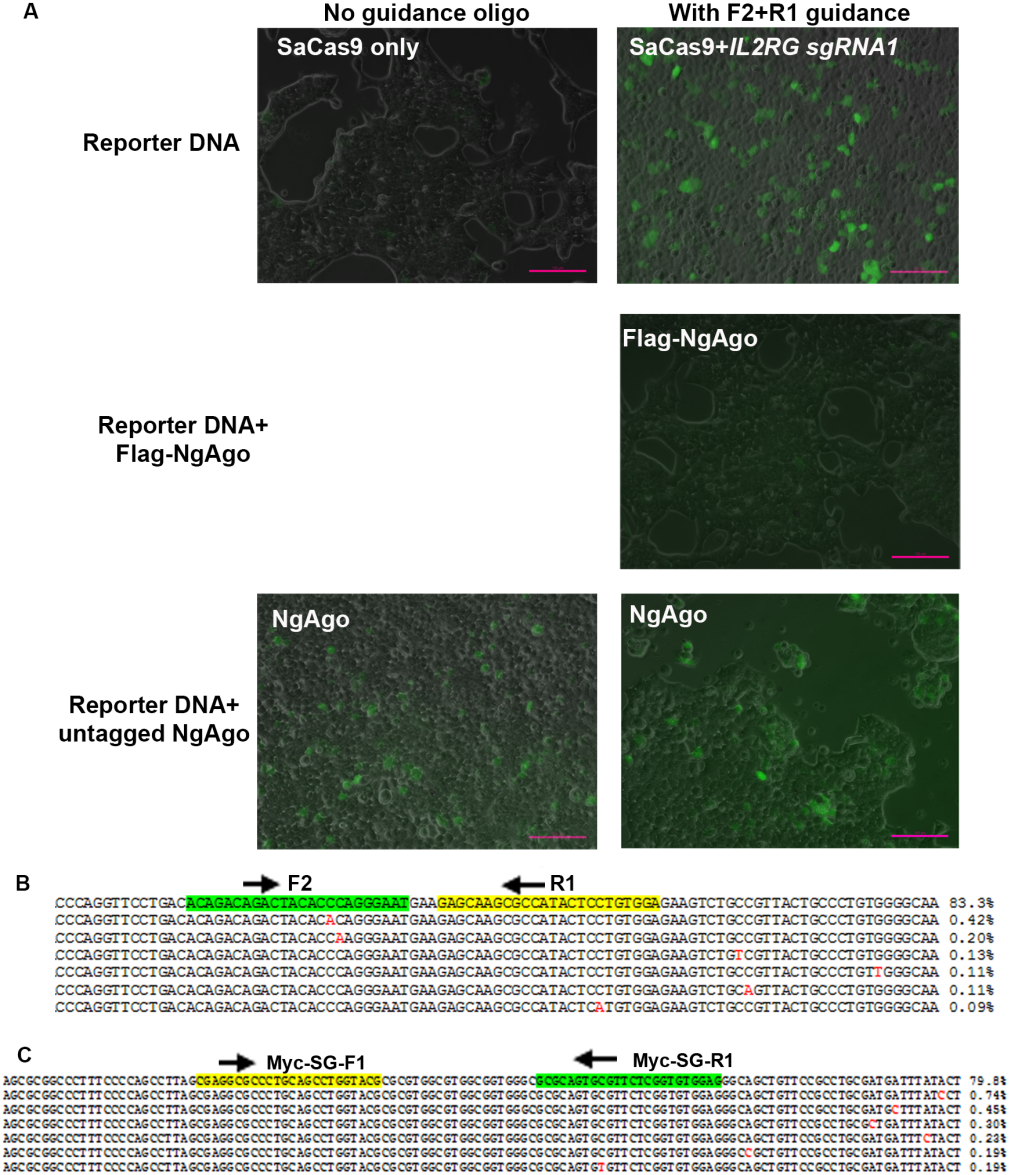
NgAgo showed no genome editing activity on plasmid and genomic DNA. **A**. Untagged NgAgo produced green fluorescence positive cells independent of guide DNA. SaCas9, tagged NgAgo or untagged NgAgo were cotransfected with reporter plasmid DNA into HEK293T cells. In SaCas9 transfection single strand guide DNA was included to equate transfection conditions. Scale bar: 100 μm. **B**. Next-generation sequencing of the target region of reporter plasmid DNA amplified from cells co-transfected with untagged NgAgo and F2+R1 oligos. The most appearing seven types of reads are listed with their percentages. Positions of the guide oligos are highlighted, with direction indicated by arrows. **C**. Sequencing of the target region of human *MYC* promoter amplified from cells co-transfected with untagged NgAgo and the indicated oligos. The most appearing seven types of reads are listed with their percentages. Positions of the guide oligos are highlighted, with direction indicated by arrows.

Since both NgAgo and TtAgo (argonaute from *Thermus thermophilus*) were reported to cleave plasmid DNA in *in vitro* cleavage assays [16,19], we wonder whether NgAgo could be functional in cells on naked DNA instead of genomic DNA with nucleosome structures. To test this possibility, the NgAgo plasmid DNA, the guide oligos, and the pSin-EF2-*IL2RG*-*HBB*-GFP reporter plasmid DNA were co-transfected into HEK293T cells. Again, pSaCas9-*IL2RG*-sgRNA1 and pSaCas9-*HBB*-sgRNA1 DNA were used as positive controls. Whereas co-transfecting pSaCas9-*IL2RG*-sgRNA1 and pSaCas9-*HBB*-sgRNA1 with the reporter DNA (single-strand guide DNA was also included in these co-transfections to ensure similar transfection conditions) produced many bright EGFP-positive cells 24 hours after transfection, co-transfecting NgAgo DNA expressing the HA- or Flag-tagged NgAgo, guide oligos and the reporter DNA produced no EGFP-positive cells, even after 72 hours (Fig 4A, middle row, images from HA-NgAgo transfected cells not shown).

Co-transfection of plasmid DNA expressing the untagged NgAgo, 5’ phosphorylated guide DNA and the reporter DNA produced appreciable numbers of cells with green fluorescence signals compared with control cells transfected with only the reporter plasmid DNA. Although the green fluorescence signal was in general weaker than that from pSaCas9-*HBB*-sgRNA1 co-transfected cells (Fig 4A, bottom right), flow cytometry detected up to 10% green fluorescence-positive cells. However, a similar percentage of green fluorescence positive cells were also observed after the co-transfection of untagged NgAgo and the reporter DNA in the absence of guide DNA (Fig 4A, bottom left). This suggested that the detected fluorescence signal was not due to NgAgo-guide DNA complex mediated genome editing.

Despite the observation of green fluorescence from NgAgo transfected cells, Western blotting with EGFP antibody failed to detect EGFP expression. To further test whether NgAgo has genome editing activity, we purified DNA from three types of NgAgo co-transfected cells: cells co-transfected with NgAgo plasmid and guide DNA F1, cells co-transfected with NgAgo plasmid with guide DNA R1 plus F2, and cells co-transfected with NgAgo plasmid but without guide DNA as a control (see Fig 1 for guide DNA location). We amplified the target region by PCR with high fidelity DNA polymerase, and sequenced the PCR products by next-generation sequencing. Similarly to our earlier results, about 85% of the reads had no mutations in all three samples; in the remainder, the 20 most appearing types of mutations were all point mutations with no correlation to the guide (Fig 4B). We reason that these point mutations are most likely the results of PCR amplification errors and erroneous calls from the next-generation sequencing. We then analyzed the percentage of reads with INDELs. In this analysis, 99.9% of the reads had no INDELs in all of the three samples. Thus, although it is unclear why we could see green fluorescence-positive cells after NgAgo and reporter co-transfection, the fluorescence signals were not the result of EGFP expression from NgAgo-generated INDELs on reporter plasmid DNA.

We further used NgAgo to target the endogenous *MYC* promoter region in addition to targeting artificial reporter sequences. The rationale for picking this region is that it is hypersensitive to DNaseI and is less packed into nucleosome structures [28], and thus should be more accessible to nucleases. We designed two 5’ phosphorylated single strand oligos (Myc-SG-F1 and Myc-SG-R1), which were 20 bp away and targeted the opposite strands of the *MYC* promoter. We co-transfected single or both oligos with DNA expressing the untagged NgAgo into HEK293T cells, amplified the target region by PCR 3 days after the transfection, and sequenced the PCR products by next-generation sequencing. We sequenced 4 samples, which all received NgAgo transfection but with different 5’ phosphorylated guide DNA: Myc-SG-F1, Myc-SG-R1, Myc-SG-F1+ Myc-SG-R1, and Myc-SG-F2+Myc-SG-R2. Both Myc-SG-F2 and Myc-SG-R2 were outside the sequenced region and thus served as background controls. For all 4 samples, 80% of readings had no mutations and the 20 most appearing types of mutations were all point mutations with no correlation to the guide (Fig 4C). Reads with INDELs were less than 0.4% for all 4 samples. Thus, our next-generation sequencing analyses found that NgAgo had no gene editing activity on naked plasmid DNA or genomic DNA.

The authors of the original NgAgo paper indicated in a protocol posted on the Addgene website (http://www.addgene.org/78253/) that NgAgo was sensitive to mycoplasma contamination. We tested our HEK293T cells and reporter cells, and both were negative for mycoplasma (data not shown).

In our hands, NgAgo did not show guide-dependent nuclease activity in HEK293T cells, whether assayed on transfected reporter plasmid DNA, integrated reporter sequences, or endogenous genes. While it is still possible that NgAgo has genome editing activity under different conditions, it is also possible that the reported *in vivo* genome editing activity of NgAgo [19] results from activities other than genome editing. Indeed, only indirect evidence was available for NgAgo’s *in vivo* genome editing activity: change of GFP expression and homologous recombination in the presence of a homologous template. For the former, altered GFP expression could be caused by fluorescence signal through an unknown, guide-independent mechanism. We observed that the untagged NgAgo increased fluorescence signal through unknown mechanisms, and was not guide-dependent. For the latter piece of evidence, homologous recombination can happen at low efficiency even without exogenous nucleases, as long as the template with homologous sequences is present. In addition, Gao et al used G418 to select for colonies in HEK293T cells [19]. To our knowledge, HEK293T cells (originally called 293tsA1609neo) should be resistant to G418, since they were created by transfection of HEK293 cells with a gene encoding the SV40 T-antigen and a neomycin resistance gene [29].

While this paper was under review, three others reported the failure to observe genome editing activity from NgAgo in various eukaryotic cells [26, 30, 31]. Ye et al further showed that NgAgo cleaves RNA rather than DNA in a guide DNA-dependent manner [32]. These results are consistent with our observation that NgAgo is unable to mediate genome editing.

In conclusion, our results and data from other researchers fail to confirm the reported DNA-guided genome editing activity of NgAgo. Current evidence suggests that NgAgo functions through mechanisms other than DNA interference. Although it seems highly unlikely that failure to observe genome editing activity from NgAgo was due to technical issues, we cannot exclude the possibility that NgAgo may only show DNA-guided genome editing activity under conditions that we have not yet found.

## Supporting information

S1 File cDNA and protein sequences of NgAgo. **Fig A.** cDNA sequence of HA-tagged NgAgo. Codons are optimized for human. Two NLS signals are included. Color code: Yellow for Tag, green for the first NLS1, pink for the second NLS2. The stop codon is underlined. The start codon is in bold font and underlined. **Fig B.** Protein sequence of HA-tagged NgAgo. Color code: Yellow for Tag, green for the first NLS1, pink for the second NLS2. **Fig C.** cDNA sequence of Flag-tagged NgAgo. Codons are optimized for human. Color code: Yellow for Tag, green for NLS. The stop codon is underlined. The start codon is in bold font and underlined. **Fig D.** Protein sequence of Flag-tagged NgAgo. Color code: Yellow for Tag, green for NLS. **Table A. Oligonucleotides used in the study.**
(DOCX)

## References

[1] Kim YG, Cha J, Chandrasegaran S (1996) Hybrid restriction enzymes: zinc finger fusions to Fok I cleavage domain. Proc Natl Acad Sci U S A 93: 1156–1160. 8577732 10.1073/pnas.93.3.1156 PMC40048

[2] Bibikova M, Beumer K, Trautman JK, Carroll D (2003) Enhancing gene targeting with designed zinc finger nucleases. Science 300: 764 10.1126/science.1079512 12730594

[3] Tebas P, Stein D, Tang WW, Frank I, Wang SQ, Lee G, et al (2014) Gene editing of CCR5 in autologous CD4 T cells of persons infected with HIV. N Engl J Med 370: 901–910. 10.1056/NEJMoa1300662 24597865 PMC4084652

[4] Cornu TI, Thibodeau-Beganny S, Guhl E, Alwin S, Eichtinger M, Joung JK, et al (2008) DNA-binding specificity is a major determinant of the activity and toxicity of zinc-finger nucleases. Mol Ther 16: 352–358. 10.1038/sj.mt.6300357 28178540

[5] Moscou MJ, Bogdanove AJ (2009) A simple cipher governs DNA recognition by TAL effectors. Science 326: 1501 10.1126/science.1178817 19933106

[6] Boch J, Scholze H, Schornack S, Landgraf A, Hahn S, Kay S, et al (2009) Breaking the code of DNA binding specificity of TAL-type III effectors. Science 326: 1509–1512. 10.1126/science.1178811 19933107

[7] Miller JC, Tan S, Qiao G, Barlow KA, Wang J, Xia DF, et al (2011) A TALE nuclease architecture for efficient genome editing. Nat Biotechnol 29: 143–148. 10.1038/nbt.1755 21179091

[8] Jinek M, Chylinski K, Fonfara I, Hauer M, Doudna JA, Charpentier E. (2012) A programmable dual-RNA-guided DNA endonuclease in adaptive bacterial immunity. Science 337: 816–821. 10.1126/science.1225829 22745249 PMC6286148

[9] Cong L, Ran FA, Cox D, Lin S, Barretto R, Habib N, et al (2013) Multiplex genome engineering using CRISPR/Cas systems. Science 339: 819–823. 10.1126/science.1231143 23287718 PMC3795411

[10] Cho SW, Kim S, Kim JM, Kim JS (2013) Targeted genome engineering in human cells with the Cas9 RNA-guided endonuclease. Nat Biotechnol 31: 230–232. 10.1038/nbt.2507 23360966

[11] Mali P, Yang L, Esvelt KM, Aach J, Guell M, DiCarlo JE, et al (2013) RNA-guided human genome engineering via Cas9. Science 339: 823–826. 10.1126/science.1232033 23287722 PMC3712628

[12] Jinek M, East A, Cheng A, Lin S, Ma E, Doudna J. (2013) RNA-programmed genome editing in human cells. Elife 2: e00471 10.7554/eLife.00471 23386978 PMC3557905

[13] Ran FA, Cong L, Yan WX, Scott DA, Gootenberg JS, et al (2015) In vivo genome editing using Staphylococcus aureus Cas9. Nature 520: 186–191. 10.1038/nature14299 25830891 PMC4393360

[14] Nelson CE, Hakim CH, Ousterout DG, Thakore PI, Moreb EA, Kriz AJ, et al (2016) In vivo genome editing improves muscle function in a mouse model of Duchenne muscular dystrophy. Science 351: 403–407. 10.1126/science.aad5143 26721684 PMC4883596

[15] Tabebordbar M, Zhu K, Cheng JK, Chew WL, Widrick JJ, Yan WX, et al (2016) In vivo gene editing in dystrophic mouse muscle and muscle stem cells. Science 351: 407–411. 10.1126/science.aad5177 26721686 PMC4924477

[16] Swarts DC, Jore MM, Westra ER, Zhu Y, Janssen JH, Snijders AP, et al (2014) DNA-guided DNA interference by a prokaryotic Argonaute. Nature 507: 258–261. 10.1038/nature12971 24531762 PMC4697943

[17] Swarts DC, Hegge JW, Hinojo I, Shiimori M, Ellis MA, Dumrongkulraksa J, et al (2015) Argonaute of the archaeon Pyrococcus furiosus is a DNA-guided nuclease that targets cognate DNA. Nucleic Acids Res 43: 5120–5129. 10.1093/nar/gkv415 25925567 PMC4446448

[18] Zander A, Holzmeister P, Klose D, Tinnefeld P, Grohmann D (2014) Single-molecule FRET supports the two-state model of Argonaute action. RNA Biol 11: 45–56. 10.4161/rna.27446 24442234 PMC3929424

[19] Gao F, Shen XZ, Jiang F, Wu Y, Han C (2016) DNA-guided genome editing using the Natronobacterium gregoryi Argonaute. Nat Biotechnol 34: 768–773. 10.1038/nbt.3547 27136078

[20] Li X, Li Y, Liu C, Jin M, Lu B (2016) Oocyte-Specific Expression of Mouse MEX3C652AA in the Ovary and Its Potential Role in Regulating Maternal Fos mRNA. Biol Reprod 94: 115 10.1095/biolreprod.115.136630 27053362

[21] Green MR, Sambrook J, MacCallum P. (2012) Molecular Cloning: A Laboratory Manual Green MR, Sambrook J, MacCallum P, editor. New York: Cold Spring Harbor Laboratory Press.

[22] Naujok O, Diekmann U, Elsner M. Gene Transfer into Pluripotent Stem Cells via Lentiviral Transduction. Methods Mol Biol. 2016;1341:67–85. 10.1007/7651_2015_221 25762298

[23] Bhattacharyya D, Hammond AT, Glick BS (2010) High-quality immunofluorescence of cultured cells. Methods Mol Biol 619: 403–410. 10.1007/978-1-60327-412-8_24 20419424 PMC2893412

[24] Cardarelli F, Digiacomo L, Marchini C, Amici A, Salomone F, Fiume G, et al (2016) The intracellular trafficking mechanism of Lipofectamine-based transfection reagents and its implication for gene delivery. Sci Rep 6: 25879 10.1038/srep25879 27165510 PMC4863168

[25] Elouahabi A, Ruysschaert JM (2005) Formation and intracellular trafficking of lipoplexes and polyplexes. Mol Ther 11: 336–347. 10.1016/j.ymthe.2004.12.006 15727930

[26] Lee SH, Turchiano G, Ata H, Nowsheen S, Romito M, Lou Z, et al (2016) Failure to detect DNA-guided genome editing using Natronobacterium gregoryi Argonaute. Nat Biotechnol 35: 17–18. 10.1038/nbt.3753 27893702 PMC5662444

[27] Yonezawa M, Otsuka T, Matsui N, Tsuji H, Kato KH, Moriyama A, et al (1996) Hyperthermia induces apoptosis in malignant fibrous histiocytoma cells in vitro. Int J Cancer 66: 347–351. 10.1002/(SICI)1097-0215(19960503)66:3<347::AID-IJC14>3.0.CO;2-8 8621256

[28] Hansel-Hertsch R, Beraldi D, Lensing SV, Marsico G, Zyner K, Parry A, et al (2016) G-quadruplex structures mark human regulatory chromatin. Nat Genet 48: 1267–72 10.1038/ng.3662 27618450

[29] DuBridge RB, Tang P, Hsia HC, Leong PM, Miller JH, Calos MP. (1987) Analysis of mutation in human cells by using an Epstein-Barr virus shuttle system. Mol Cell Biol 7: 379–387. 3031469 10.1128/mcb.7.1.379-387.1987 PMC365079

[30] Burgess S, Cheng L, Gu F, Huang J, Huang Z, Lin S, et al (2016) Questions about NgAgo. Protein Cell 7: 913–915. 10.1007/s13238-016-0343-9 27848216 PMC5205665

[31] Qi J, Dong Z, Shi Y, Wang X, Qin Y, Wang Y, et al (2016) NgAgo-based fabp11a gene knockdown causes eye developmental defects in zebrafish. Cell Res 26: 1349–1352. 10.1038/cr.2016.134 27834346 PMC5143420

[32] Ye S, Bae T, Kim K, Habib O, Lee SH, Kim YY, et al (2017) DNA-dependent RNA cleavage by the Natronobacterium gregoryi Argonaute. BioRxiv. 10.1101/101923

