# Supplementary material for: No evidence of genome editing activity from Natronobacterium gregoryi Argonaute (NgAgo) in human cells
Source: PLoS One. 2017 May 11;12(5):e0177444. doi: 10.1371/journal.pone.0177444 (PMC5426773; doi:10.1371/journal.pone.0177444)
Supplement: S1 File — Fig A. cDNA sequence of HA-tagged NgAgo. Codons are optimized for human. Two NLS signals are included. Color code: Yellow for Tag, green for the first NLS1, pink for the second NLS2. The stop codon is underlined. The start codon is in bold font and underlined. Fig B. Protein sequence of HA-tagged NgAgo. Color code: Yellow for Tag, green for the first NLS1, pink for the second NLS2. Fig C. cDNA sequence of Flag-tagged NgAgo. Codons are optimized for human. Color code: Yellow for Tag, green for NLS. The stop codon is underlined. The start codon is in bold font and underlined. Fig D. Protein sequence of Flag-tagged NgAgo. Color code: Yellow for Tag, green for NLS. Table A. Oligonucleotides used in the study. (DOCX) [file pone.0177444.s001.docx]

**S1 File. cDNA and protein sequences of NgAgo.**

**Fig A. cDNA sequence of HA-tagged NgAgo.** Codons are optimized for human. Two NLS signals are included. Color code: Yellow for Tag, green for the first NLS1, pink for the second NLS2. The stop codon is underlined. The start codon is in bold font and underlined.

CATCGAT***GCCACC***

**ATG** GCG TAC CCC TAC GAC GTA CCG GAC TAT GCA CCA AAG AAA AAA CGG AAA GTT GGT GGT GGA TCC AAA AGA CCC GCC GCT ACC AAG AAG GCG GGG CAG GCA AAA AAG AAG AAG GGT ACC GGA GGA ATG ACA GTG ATA GAC CTG GAC TCA ACT ACA ACG GCC GAC GAG CTG ACA AGC GGC CAT ACT TAT GAC ATA AGC GTC ACA CTG ACT GGC GTC TAT GAC AAC ACT GAT GAG CAA CAC CCC CGG ATG TCT CTC GCA TTT GAG CAG GAT AAT GGT GAG AGA CGA TAT ATA ACC CTC TGG AAG AAT ACG ACG CCT AAG GAT GTA TTT ACG TAT GAT TAC GCA ACG GGA AGT ACC TAT ATT TTC ACA AAC ATT GAT TAC GAA GTT AAG GAT GGT TAC GAG AAT CTG ACT GCA ACG TAT CAG ACG ACG GTC GAG AAC GCT ACT GCA CAG GAG GTA GGG ACT ACG GAT GAG GAC GAG ACC TTC GCT GGC GGG GAG CCC CTG GAT CAT CAT CTG GAT GAT GCC TTG AAT GAG ACG CCG GAC GAC GCA GAG ACA GAA TCA GAC AGT GGC CAT GTG ATG ACC TCA TTT GCA TCA AGG GAC CAG CTG CCG GAG TGG ACC CTC CAC ACA TAC ACA CTC ACC GCA ACG GAT GGG GCC AAA ACT GAC ACG GAG TAC GCA CGG CGA ACG CTG GCT TAT ACC GTG CGG CAG GAG CTC TAT ACC GAC CAT GAT GCG GCG CCA GTT GCG ACT GAC GGT CTG ATG CTT TTG ACC CCA GAA CCT CTC GGG GAG ACT CCC CTT GAC CTC GAC TGT GGC GTT CGC GTA GAG GCT GAC GAA ACG AGG ACT CTT GAT TAC ACT ACA GCT AAG GAC CGC CTC CTT GCA CGA GAG TTG GTG GAA GAA GGC TTG AAG CGG TCC TTG TGG GAC GAC TAT CTC GTC CGG GGG ATT GAT GAA GTA CTG AGT AAA GAA CCC GTG CTC ACC TGC GAC GAA TTT GAC CTC CAC GAA CGA TAT GAC CTC TCC GTC GAG GTG GGC CAT TCA GGA CGA GCT TAT CTG CAT ATT AAT TTC CGA CAT CGC TTC GTG CCC AAG TTG ACA CTC GCC GAC ATC GAC GAT GAT AAC ATC TAT CCA GGT TTG CGG GTC AAA ACC ACA TAT AGA CCA AGG CGA GGA CAC ATC GTC TGG GGC CTC AGA GAC GAA TGT GCA ACA GAT AGC CTC AAT ACC CTT GGG AAT CAG TCC GTC GTG GCC TAT CAT CGA AAT AAT CAA ACC CCC ATT AAC ACT GAC CTG CTG GAT GCA ATT GAG GCG GCG GAC AGA AGA GTA GTC GAA ACG CGC AGA CAG GGT CAT GGA GAT GAT GCA GTG TCT TTT CCG CAA GAG CTT CTG GCG GTG GAG CCA AAC ACC CAT CAG ATT AAG CAG TTC GCG AGT GAC GGG TTC CAT CAA CAG GCC CGC AGC AAG ACA CGA TTG AGC GCT AGT AGG TGT TCC GAA AAG GCC CAA GCC TTT GCC GAA CGC CTC GAC CCA GTG CGA CTG AAC GGA AGT ACC GTC GAG TTT AGC AGT GAG TTC TTC ACA GGG AAT AAC GAG CAG CAG CTT CGC CTG TTG TAT GAA AAT GGT GAG TCC GTG TTG ACA TTC CGC GAT GGT GCC AGA GGA GCG CAC CCC GAC GAG ACG TTT TCA AAG GGC ATC GTG AAC CCG CCC GAG AGC TTT GAA GTG GCT GTT GTC TTG CCG GAG CAA CAG GCG GAT ACG TGC AAA GCG CAG TGG GAT ACT ATG GCC GAC CTG CTT AAC CAG GCC GGG GCT CCC CCT ACG AGA AGC GAG ACG GTC CAA TAT GAT GCC TTT TCT AGC CCA GAG TCA ATC AGC CTC AAC GTA GCT GGA GCG ATA GAC CCA AGT GAG GTT GAT GCA GCG TTC GTT GTA CTG CCA CCC GAC CAG GAG GGC TTC GCC GAT CTG GCT TCC CCT ACG GAG ACG TAT GAT GAA TTG AAG AAG GCA TTG GCG AAC ATG GGG ATC TAT TCT CAA ATG GCA TAC TTC GAC AGG TTT AGA GAC GCA AAA ATT TTC TAT ACG CGA AAC GTA GCC CTT GGC CTG CTT GCT GCA GCA GGC GGT GTC GCA TTT ACA ACA GAG CAT GCG ATG CCC GGG GAC GCG GAC ATG TTC ATT GGC ATT GAC GTG AGT AGG TCT TAC CCA GAA GAC GGG GCC TCA GGG CAG ATA AAT ATT GCG GCT ACG GCG ACT GCC GTC TAC AAG GAT GGC ACT ATT CTC GGG CAC TCC AGT ACG CGG CCT CAG CTT GGT GAA AAA CTG CAG TCT ACG GAC GTA CGC GAC ATT ATG AAG AAT GCA ATC CTC GGA TAC CAA CAA GTG ACG GGC GAG TCC CCT ACC CAC ATC GTG ATT CAT AGA GAC GGT TTT ATG AAC GAG GAC CTG GAC CCT GCG ACT GAG TTT CTC AAT GAG CAA GGC GTG GAG TAT GAT ATC GTA GAG ATT AGG AAA CAG CCG CAG ACT AGA CTC CTT GCC GTC AGT GAT GTC CAG TAC GAC ACA CCG GTA AAA TCA ATT GCC GCC ATC AAC CAA AAT GAA CCG AGG GCC ACG GTA GCC ACT TTC GGG GCC CCC GAG TAC TTG GCG ACC AGG GAT GGT GGA GGA TTG CCC AGG CCA ATT CAA ATC GAA AGA GTG GCC GGT GAA ACC GAT ATA GAA ACA CTG ACT AGA CAA GTT TAT CTC CTC TCA CAG AGC CAT ATT CAG GTG CAC AAC TCC ACT GCT CGG TTG CCT ATT ACT ACG GCC TAC GCA GAC CAA GCT TCT ACA CAT GCG ACA AAG GGA TAT CTG GTG CAA ACT GGT GCC TTT GAG TCA AAC GTA GGG TTC TTG TGA GATCT

**Fig B. Protein sequence of HA-tagged NgAgo.** Color code: Yellow for Tag, green for the first NLS1, pink for the second NLS2.

MAYPYDVPDYAPKKKRKVGGGsKRPAATKKAGQAKKKKGtGGmtvidldstttadeltsghtydisvtltgvydntdeqhprmslafeqdngerryitlwknttpkdvftydyatgstyiftnidyevkdgyenltatyqttvenataqevgttdedetfaggepldhhlddalnetpddaetesdsghvmtsfasrdqlpewtlhtytltatdgaktdteyarrtlaytvrqelytdhdaapvatdglmlltpeplgetpldldcgvrveadetrtldyttakdrllarelveeglkrslwddylvrgidevlskepvltcdefdlherydlsvevghsgraylhinfrhrfvpkltladidddniypglrvkttyrprrghivwglrdecatdslntlgnqsvvayhrnnqtpintdlldaieaadrrvvetrrqghgddavsfpqellavepnthqikqfasdgfhqqarsktrlsasrcsekaqafaerldpvrlngstvefssefftgnneqqlrllyengesvltfrdgargahpdetfskgivnppesfevavvlpeqqadtckaqwdtmadllnqagapptrsetvqydafsspesislnvagaidpsevdaafvvlppdqegfadlasptetydelkkalanmgiysqmayfdrfrdakifytrnvalgllaaaggvafttehampgdadmfigidvsrsypedgasgqiniaatatavykdgtilghsstrpqlgeklqstdvrdimknailgyqqvtgespthivihrdgfmnedldpateflneqgveydiveirkqpqtrllavsdvqydtpvksiaainqnepratvatfgapeylatrdggglprpiqiervagetdietltrqvyllsqshiqvhnstarlpittayadqasthatkgylvqtgafesnvgfl (929AA)

**Fig C. cDNA sequence of Flag-tagged NgAgo.** Codons are optimized for human. Color code: Yellow for Tag, green for NLS. The stop codon is underlined. The start codon is in bold font and underlined.

catcgaattc*acc* **ATG**GAC TAC AAA GAC GAT GAC GAC AAG GCT CCA AAG AAA AAA CGG AAA GTT GGT ACC

GGA GGA

ATG ACA GTG ATA GAC CTG GAC TCA ACT ACA ACG GCC GAC GAG CTG ACA AGC GGC CAT ACT TAT GAC ATA AGC GTC ACA CTG ACT GGC GTC TAT GAC AAC ACT GAT GAG CAA CAC CCC CGG ATG TCT CTC GCA TTT GAG CAG GAT AAT GGT GAG AGA CGA TAT ATA ACC CTC TGG AAG AAT ACG ACG CCT AAG GAT GTA TTT ACG TAT GAT TAC GCA ACG GGA AGT ACC TAT ATT TTC ACA AAC ATT GAT TAC GAA GTT AAG GAT GGT TAC GAG AAT CTG ACT GCA ACG TAT CAG ACG ACG GTC GAG AAC GCT ACT GCA CAG GAG GTA GGG ACT ACG GAT GAG GAC GAG ACC TTC GCT GGC GGG GAG CCC CTG GAT CAT CAT CTG GAT GAT GCC TTG AAT GAG ACG CCG GAC GAC GCA GAG ACA GAA TCA GAC AGT GGC CAT GTG ATG ACC TCA TTT GCA TCA AGG GAC CAG CTG CCG GAG TGG ACC CTC CAC ACA TAC ACA CTC ACC GCA ACG GAT GGG GCC AAA ACT GAC ACG GAG TAC GCA CGG CGA ACG CTG GCT TAT ACC GTG CGG CAG GAG CTC TAT ACC GAC CAT GAT GCG GCG CCA GTT GCG ACT GAC GGT CTG ATG CTT TTG ACC CCA GAA CCT CTC GGG GAG ACT CCC CTT GAC CTC GAC TGT GGC GTT CGC GTA GAG GCT GAC GAA ACG AGG ACT CTT GAT TAC ACT ACA GCT AAG GAC CGC CTC CTT GCA CGA GAG TTG GTG GAA GAA GGC TTG AAG CGG TCC TTG TGG GAC GAC TAT CTC GTC CGG GGG ATT GAT GAA GTA CTG AGT AAA GAA CCC GTG CTC ACC TGC GAC GAA TTT GAC CTC CAC GAA CGA TAT GAC CTC TCC GTC GAG GTG GGC CAT TCA GGA CGA GCT TAT CTG CAT ATT AAT TTC CGA CAT CGC TTC GTG CCC AAG TTG ACA CTC GCC GAC ATC GAC GAT GAT AAC ATC TAT CCA GGT TTG CGG GTC AAA ACC ACA TAT AGA CCA AGG CGA GGA CAC ATC GTC TGG GGC CTC AGA GAC GAA TGT GCA ACA GAT AGC CTC AAT ACC CTT GGG AAT CAG TCC GTC GTG GCC TAT CAT CGA AAT AAT CAA ACC CCC ATT AAC ACT GAC CTG CTG GAT GCA ATT GAG GCG GCG GAC AGA AGA GTA GTC GAA ACG CGC AGA CAG GGT CAT GGA GAT GAT GCA GTG TCT TTT CCG CAA GAG CTT CTG GCG GTG GAG CCA AAC ACC CAT CAG ATT AAG CAG TTC GCG AGT GAC GGG TTC CAT CAA CAG GCC CGC AGC AAG ACA CGA TTG AGC GCT AGT AGG TGT TCC GAA AAG GCC CAA GCC TTT GCC GAA CGC CTC GAC CCA GTG CGA CTG AAC GGA AGT ACC GTC GAG TTT AGC AGT GAG TTC TTC ACA GGG AAT AAC GAG CAG CAG CTT CGC CTG TTG TAT GAA AAT GGT GAG TCC GTG TTG ACA TTC CGC GAT GGT GCC AGA GGA GCG CAC CCC GAC GAG ACG TTT TCA AAG GGC ATC GTG AAC CCG CCC GAG AGC TTT GAA GTG GCT GTT GTC TTG CCG GAG CAA CAG GCG GAT ACG TGC AAA GCG CAG TGG GAT ACT ATG GCC GAC CTG CTT AAC CAG GCC GGG GCT CCC CCT ACG AGA AGC GAG ACG GTC CAA TAT GAT GCC TTT TCT AGC CCA GAG TCA ATC AGC CTC AAC GTA GCT GGA GCG ATA GAC CCA AGT GAG GTT GAT GCA GCG TTC GTT GTA CTG CCA CCC GAC CAG GAG GGC TTC GCC GAT CTG GCT TCC CCT ACG GAG ACG TAT GAT GAA TTG AAG AAG GCA TTG GCG AAC ATG GGG ATC TAT TCT CAA ATG GCA TAC TTC GAC AGG TTT AGA GAC GCA AAA ATT TTC TAT ACG CGA AAC GTA GCC CTT GGC CTG CTT GCT GCA GCA GGC GGT GTC GCA TTT ACA ACA GAG CAT GCG ATG CCC GGG GAC GCG GAC ATG TTC ATT GGC ATT GAC GTG AGT AGG TCT TAC CCA GAA GAC GGG GCC TCA GGG CAG ATA AAT ATT GCG GCT ACG GCG ACT GCC GTC TAC AAG GAT GGC ACT ATT CTC GGG CAC TCC AGT ACG CGG CCT CAG CTT GGT GAA AAA CTG CAG TCT ACG GAC GTA CGC GAC ATT ATG AAG AAT GCA ATC CTC GGA TAC CAA CAA GTG ACG GGC GAG TCC CCT ACC CAC ATC GTG ATT CAT AGA GAC GGT TTT ATG AAC GAG GAC CTG GAC CCT GCG ACT GAG TTT CTC AAT GAG CAA GGC GTG GAG TAT GAT ATC GTA GAG ATT AGG AAA CAG CCG CAG ACT AGA CTC CTT GCC GTC AGT GAT GTC CAG TAC GAC ACA CCG GTA AAA TCA ATT GCC GCC ATC AAC CAA AAT GAA CCG AGG GCC ACG GTA GCC ACT TTC GGG GCC CCC GAG TAC TTG GCG ACC AGG GAT GGT GGA GGA TTG CCC AGG CCA ATT CAA ATC GAA AGA GTG GCC GGT GAA ACC GAT ATA GAA ACA CTG ACT AGA CAA GTT TAT CTC CTC TCA CAG AGC CAT ATT CAG GTG CAC AAC TCC ACT GCT CGG TTG CCT ATT ACT ACG GCC TAC GCA GAC CAA GCT TCT ACA CAT GCG ACA AAG GGA TAT CTG GTG CAA ACT GGT GCC TTT GAG TCA AAC GTA GGG TTC TTG TGA GATCT

**Fig D. Protein sequence of Flag-tagged NgAgo.** Color code: Yellow for Tag, green for NLS.

MDYKDDDDKAPKKKRKVGTGGMTVIDLDSTTTADELTSGHTYDISVTLTGVYDNTDEQHPRMSLAFEQDNGERRYITLWKNTTPKDVFTYDYATGSTYIFTNIDYEVKDGYENLTATYQTTVENATAQEVGTTDEDETFAGGEPLDHHLDDALNETPDDAETESDSGHVMTSFASRDQLPEWTLHTYTLTATDGAKTDTEYARRTLAYTVRQELYTDHDAAPVATDGLMLLTPEPLGETPLDLDCGVRVEADETRTLDYTTAKDRLLARELVEEGLKRSLWDDYLVRGIDEVLSKEPVLTCDEFDLHERYDLSVEVGHSGRAYLHINFRHRFVPKLTLADIDDDNIYPGLRVKTTYRPRRGHIVWGLRDECATDSLNTLGNQSVVAYHRNNQTPINTDLLDAIEAADRRVVETRRQGHGDDAVSFPQELLAVEPNTHQIKQFASDGFHQQARSKTRLSASRCSEKAQAFAERLDPVRLNGSTVEFSSEFFTGNNEQQLRLLYENGESVLTFRDGARGAHPDETFSKGIVNPPESFEVAVVLPEQQADTCKAQWDTMADLLNQAGAPPTRSETVQYDAFSSPESISLNVAGAIDPSEVDAAFVVLPPDQEGFADLASPTETYDELKKALANMGIYSQMAYFDRFRDAKIFYTRNVALGLLAAAGGVAFTTEHAMPGDADMFIGIDVSRSYPEDGASGQINIAATATAVYKDGTILGHSSTRPQLGEKLQSTDVRDIMKNAILGYQQVTGESPTHIVIHRDGFMNEDLDPATEFLNEQGVEYDIVEIRKQPQTRLLAVSDVQYDTPVKSIAAINQNEPRATVATFGAPEYLATRDGGGLPRPIQIERVAGETDIETLTRQVYLLSQSHIQVHNSTARLPITTAYADQASTHATKGYLVQTGAFESNVGFL

**Table A. Oligonucleotides used in the study**

| Primer name | Sequence | Purpose |
| --- | --- | --- |
| HBB-F | GTGTTCACTAGCAACCTCAAACAG | As primers to amplify human *HBB* target region for next-generation sequencing |
| HBB-R | CAATAGGCAGAGAGAGTCAGTG |  |
| IL2RG-F | TGAAGCTATGACAGAGGAAACG | As primers to amplify human *IL2RG* target region for next-generation sequencing |
| IL2RG-R | GCAGCTGCAGGAATAAGAGG |  |
| Myc-F | CACTCTCCCTGGGACTCTTG | As primers to amplify human *MYC* target region for next-generation sequencing |
| Myc-R | TCTCCCTTTCTCTGCTGCTC |  |
| Reporter-F | TCCATTTCAGGTGTCGTGAG | As primers to amplify the *IL2RG* and *HBB* target sequence from the reporter plasmid for next-generation sequencing |
| Reporter-R1 | GAACTTCAGGGTCAGCTTGC |  |
| Myc4G-SG-F1 | (5’phos)CGAGGCGCCCTGCAGCCTGGTACG | As guide DNA for *MYC* promoter region |
| Myc4G-SG-R1 | (5’phos)CTCCACACCGAGAACGCACTGCGC | As guide DNA for *MYC* promoter region |
| Myc4G-SG-F2 | (5’phos)CCTTCCCCACCCTCCCCACCCTCC | As guide DNA for *MYC* promoter region |
| Myc4G-SG-R2 | GGAGGGTGGGGAGGGTGGGGAAGG | As guide DNA for *MYC* promoter region |
| HBB-SG-F  (F3 in Fig.1A) | (5'phos)GTCTGCCGTTACTGCCCTGTGGGG | As guide DNA for *HBB* target region (contains the whole *HBB* target sequence for SaCas9). |
| HHB-SG-R  (R3 in Fig.1A) | (5’phos)CCCCACAGGGCAGTAACGGCAGAC | As guide DNA for *HBB* target region (reverse complementary to HBB-SG-F). |
| IL2RG-SG-F  (F1 in Fig.1A) | (5'phos)ACAGACAGACTACACCCAGGGAAT | As guide DNA for *IL2RG* target region (contains the whole SaCas9 target sequence for *IL2RG*). |
| IL2RG-SG-R  (R1 in Fig.1A) | (5’phos) ATTCCCTGGGTGTAGTCTGTCTGT | As guide DNA for *IL2RG* target region (reverse complementary to IL2RG-SG-F) |
| HALF-SG-F  (F2 in Fig.1A) | (5’phos) gagcaagcgccatACTCCTGTGGA | As guide DNA for target sequence in the EGFP reporter construct, half matches *HBB* and half matches *IL2RG* target region. |
| IL2RG-g1F: | CACCGTAGTCTGTCTGTGTCAGGAAC | Annealed and inserted into pX601-AAV-CMV::NLS-SaCas9-NLS-3xHA-bGHpA;U6::BsaI-sgRNA, to express SaCas9 and *IL2RG* sgRNA 1 (pSaCas9-*IL2RG*-sgRNA 1). |
| IL2RG-g1R: | AAACGTTCCTGACACAGACAGACTAC |  |
| HBB-g1F: | CACCGAGTAACGGCAGACTTCTCCAC | Annealed and inserted into pX601-AAV-CMV::NLS-SaCas9-NLS-3xHA-bGHpA;U6::BsaI-sgRNA, to express SaCas9 and *HBB* sgRNA 1 (pSaCas9-*HHB*-sgRNA 1). |
| HBB-g1R: | AAACGTGGAGAAGTCTGCCGTTACTC |  |
| Alexa488-Luci | 5’ Alexa488-AGATGGATTCCAATTCAGCG | Fluorescence labeled oligonucleotide to check transfection efficiency. |
